# Use of a Mobile Lifestyle Intervention App as an Early Intervention for Adolescents With Obesity: Single-Cohort Study

**DOI:** 10.2196/20520

**Published:** 2021-09-28

**Authors:** Chu Shan Elaine Chew, Courtney Davis, Jie Kai Ethel Lim, Chee Meng Micheal Lim, Yi Zhen Henny Tan, Jean Yin Oh, Kumudhini Rajasegaran, Yong Hwa Michael Chia, Eric Andrew Finkelstein

**Affiliations:** 1 KK Women's and Children's Hospital Singapore Singapore; 2 ReMark Group Singapore Singapore; 3 National Institute of Education Singapore Singapore; 4 Duke-NUS Medical School Health Services and Systems Research Singapore Singapore

**Keywords:** pediatric obesity, mobile health, apps, health behavior, mHealth, obesity, adolescent, lifestyle, well-being, mobile phone

## Abstract

**Background:**

Effective, resource-efficient treatment is urgently needed to address the high rates of pediatric and adolescent obesity. This need has been accelerated by the COVID-19 pandemic. The use of a mobile health tool as an early intervention before a clinic-based multidisciplinary weight management program could be an effective treatment strategy that is appropriate during a pandemic.

**Objective:**

This study aims to assess the effectiveness of and adolescent engagement with a mobile app–based lifestyle intervention program as an early intervention before enrollment in a clinic-based multidisciplinary weight management program.

**Methods:**

This prospective single-cohort study involved adolescents, aged 10-16 years, who were overweight and obese (defined as BMI percentile above the 85th percentile). Participants used the mobile Kurbo app as an early intervention before enrolling in a clinic-based multidisciplinary weight management program. Kurbo’s health coaches provided weekly individual coaching informed by a model of supportive accountability via video chat, and participants self-monitored their health behavior. The implementation of Kurbo as an early intervention was evaluated using the reach, effectiveness, adoption, implementation, and maintenance framework by reach (number who consented to participate out of all patients approached), implementation (Kurbo engagement and evaluation), and effectiveness as measured by the primary outcome of the BMI z-score at 3 months. Secondary outcome measures included changes in body fat percentage, nutrition and physical activity levels, and quality of life at 3 months. Maintenance was defined as the outcome measures at 6-month follow-up.

**Results:**

Of the 73 adolescents who were approached for enrollment, 40 (55%) of adolescents were recruited. The mean age was 13.8 (SD 1.7) years, and the mean BMI z-score was 2.07 (SD 0.30). In the multiethnic Asian sample, 83% (33/40) of the participants had household incomes below the national median. Kurbo engagement was high, with 83% (33/40) of participants completing at least 7 coaching sessions. In total, 78% (18/23) of participants rated the app as good to excellent and 70% (16/23) stated that they would recommend it to others. There were no statistically significant changes in BMI z-scores at 3 months (*P*=.19) or 6 months *(P*=.27). Participants showed statistically significant improvements in measured body fat percentage, self-reported quality of life, and self-reported caloric intake from the 3-day food diaries at 3 and 6 months.

**Conclusions:**

The use of Kurbo before enrollment in an outpatient multidisciplinary clinical care intervention is a feasible strategy to expand the reach of adolescent obesity management services to a low-income and racially diverse population. Although there was no significant change in BMI z-scores, the use of Kurbo as an early intervention could help to improve quality of life and reduce body fat percentage and total caloric intake.

## Introduction

### Background

Mirroring global trends, the prevalence of overweight among Singaporean adolescents increased from 2.2% in 1975 to 15.9% in 2016. Overweight adolescents are at a higher risk for adult obesity as well as short- and long-term medical and psychosocial complications [1]. To address this concern in a resource-efficient manner, experts have proposed a staged care approach for the management of adolescents who are overweight and obese. Those who are less successful in reaching a healthy weight through less-intensive interventions, such as mobile health (mHealth) apps [2], are then recommended for higher stage interventions. mHealth apps have the potential for broad reach and adoption among Singaporean youth because of their low cost, widespread internet availability, and high levels of smartphone ownership [3-5]. Given the current COVID-19 pandemic, mHealth is a timely and essential tool to engage adolescents when in-person services are unavailable and when COVID-19 response strategies contribute to decreased physical activity and increase other unhealthy lifestyle behaviors [6,7,8].

The commercially available mHealth weight management program, Kurbo, has provided individualized health coaching, educational videos on nutrition and physical activity, and self-monitoring to improve diet and physical activity behaviors [9]. Prior research among Kurbo users showed that increased engagement with the Kurbo features, including the web-based coaching sessions, was associated with greater weight loss, suggesting that Kurbo may be appropriate as an early-stage intervention among adolescents with obesity [10].

### Objectives

Therefore, the primary objective of this study is to examine the implementation of Kurbo as an early intervention for adolescents with obesity before enrollment in a clinic-based multidisciplinary weight management program in Singapore. The evaluation used the relevant dimensions of the reach, effectiveness, adoption, implementation, and maintenance evaluation framework [11].

## Methods

### Study Design

This was a prospective, single-arm study that conducted evaluations at three time points: baseline, 3 months, and 6 months. A total of 40 participants, with informed parental consent and child assent, were enrolled in the study at the point of referral to the KK Women’s and Children’s Hospital (KKH) weight management clinic (WMC). Participants were enrolled between October 2018 and March 2019. All study procedures were approved by the Singhealth Centralized Institutional Review Board. The study was registered at ClinicalTrials.gov (NCT03561597).

### Multidisciplinary WMC Clinic

KKH is an 830-bed tertiary pediatric teaching hospital that provides two-third of the government-subsidized pediatric care in Singapore. The adolescent WMC is a physician-led multidisciplinary clinic where adolescents with overweight and their families engage with a multidisciplinary care team consisting of physicians, dietitians, exercise physiologists, and psychologists to set and monitor behavioral goals to manage obesity-related comorbidities. The KKH WMC protocols and outcomes have been previously published, with a historical dropout rate of 58% [12].

The usual waiting time for a first visit to the WMC clinic varies from 4 to 8 weeks after the initial referral. For this study, WMC providers had access to information about participants’ Kurbo progress through an administrator site during this period. This allowed for monitoring safety concerns and guiding discussions during clinic visits. After the first WMC visit, dietary recommendations and counseling were provided by the dietitian according to the recommended nutritional guidelines. Physical activity counseling was also performed by exercise trainers based on the World Health Organization guidelines on physical activity and sedentary behavior.

### Study Participants

Adolescents, referred to the WMC, aged 10-17 years with a BMI percentile above the 85th percentile [13], were eligible for enrollment. Adolescents with secondary causes of obesity, such as Cushing syndrome, those whose parents were non-English speakers, and those without smartphone access were not eligible for enrollment. Study participants were asked to download the Kurbo app onto their mobile phones and enroll in the app at the point of study enrollment. The participants had free access to the Kurbo program.

### Kurbo Program

**Figure 1:**
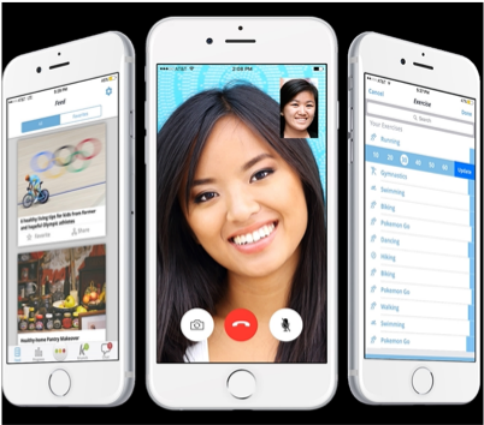
Screenshot of Kurbo app.

Kurbo is a mobile app developed to aid adolescents and their families with weight management through dietary self-monitoring and weekly coaching sessions (Figure 1). The details of the Kurbo mobile app and use of supportive accountability in the coaching program have been described previously [10,14]. In short, the Kurbo program consists of a mobile app for self-monitoring of eating, physical activity behaviors, and weight and individualized coaching sessions by Kurbo-certified behavioral coaches. The mobile app’s data-driven platform provides users with feedback via push notifications, text messages, and emails. Using the traffic light diet to categorize foods [15], Kurbo promotes the gradual reduction of high-calorie (red) foods over time. Study participants were asked to use the mobile app to log their daily food intake and were encouraged to gradually reduce their *red* food consumption and increase consumption of *green* food. They were also recommended to monitor their daily physical activity levels and to work toward the recommended 60 minutes of moderate-to-vigorous physical activity (MVPA) each day. Weight tracking and uploading into the app were recommended at least weekly. Individualized coaching included a weekly check-in with the coach for 15 minutes via video, phone, or text over a 12-week period. Participants were paired with the same coach for the duration of the program. Coaches monitored participants’ self-report of weight, physical activity, and dietary behaviors and provided individualized feedback based on the information provided. After each coaching session, coaches emailed a copy of the session summary and a tailored plan for the coming week.

### Measures

Demographic and parental characteristics were obtained at the baseline. Questionnaires and food diaries were obtained through self-reports using physical forms during the study visits.

### Evaluation

The implementation of the Kurbo program as an early intervention was evaluated using the relevant dimensions of reach, effectiveness, adoption, implementation, and maintenance framework [11] as follows: (1) reach, number who consented to participate out of all patients approached; (2) implementation, program engagement as detailed for the 12-week program and program evaluation; (3) effectiveness, measured by 3-month BMI *z*-score changes from baseline and changes in blood pressure, nutritional intake physical activity levels, quality of life, and disordered eating behaviors as defined below; and (4) maintenance, measured using the identical outcomes at 6 months and 3 months after the program conclusion. Throughout the 12-week program, we also recorded the weekly frequency of information entered for meal consumption, body weight, physical activity, and coaching sessions. We evaluated satisfaction with the Kurbo program through solicitation of participants’ feedback on ease of program navigation and overall use as well as their likelihood to recommend Kurbo to others.

### Anthropometric and Blood Pressure Measurements

Height, weight, waist circumference [16], body fat percentage [17], and blood pressure were measured by trained staff at each study visit in the clinic. Height was measured to the nearest 0.1 cm via a stadiometer (Seca, Model 220). Weight was measured to the nearest 0.1 kg using a medical weighing scale without shoes and in light clothing. Body fat percentage was assessed using bioimpedance analysis (Impedimed, DF50 Body Composition Analyser). Blood pressure was measured using an electronic sphygmomanometer (Dinamap model 8101, Critikon Inc). Anthropometric measurements were taken at baseline and at 3 and 6 months.

### Nutritional Intake

Adolescents’ daily total caloric intake and fruit and vegetable consumption were assessed using a 3-day food diary that has been previously validated for use with Singaporean adolescents [18,19]. The food diary was administered at baseline and at 3 and 6 months.

### Physical Activity Levels

Physical activity was assessed using a wGT3X+ ActiGraph accelerometer. Participants wore the accelerometer for a 7-day period at baseline, 3 months, and 6 months. The ActiGraph data were processed using ActiLife 6 software. The Puyau cutoff point of 3200 counts per minute was used to estimate the time spent in MVPA. When 20 minutes of consecutive zeros were present in the accelerometry data, it was assumed that the monitor was not being worn at that time. All days with >500 minutes of valid data were included in the analysis [20-22].

### Psychosocial Outcomes

Self-reported questionnaires were administered to adolescents at baseline, 3 months, and 6 months. The Pediatric Quality of Life Inventory (PedsQoL; UK version 4) was administered to evaluate physical, emotional, school, and social functioning. The Eating Pattern Inventory for Children (EPI-C) [23] was used to assess four dimensions of psychological eating behavior: external eating, emotional eating, dietary restraint, and parental pressure to eat. Higher scores were indicative of concerning behavior in each dimension. Both the PedsQoL and EPI-C have been validated in adolescent cohorts, including overweight adolescents [24,25].

### Statistical Analysis

Data were analyzed using SPSS version 9.3 for Windows (IBM Corp). BMI was computed as kg/m^2^, and the BMI z-score was calculated using the L, M, and S parameters published by the Centers for Disease Control and Prevention [13]. Baseline demographic, anthropometric measurements, and parent characteristics were compared between study completers and those who dropped out of the study using the Wilcoxon rank-sum test and two-sample, 2-tailed *t* tests for nonnormal and normal continuous variables, respectively, and the Fisher exact test for categorical variables. Changes in anthropometric measurements, quality of life, disordered eating behaviors, MVPA, total caloric intake, and blood pressure measurements between the first visit and months 3 and 6 were compared using paired-sample *t* tests. Linear regression was used to determine the association between BMI z-scores at 3 months (primary outcome) and the number of WMC visits attended during the Kurbo program. The level of statistical significance was set at *P*<.05.

## Results

### Implementation of Kurbo Program as an Early Intervention

#### Reach

**Figure 2:**
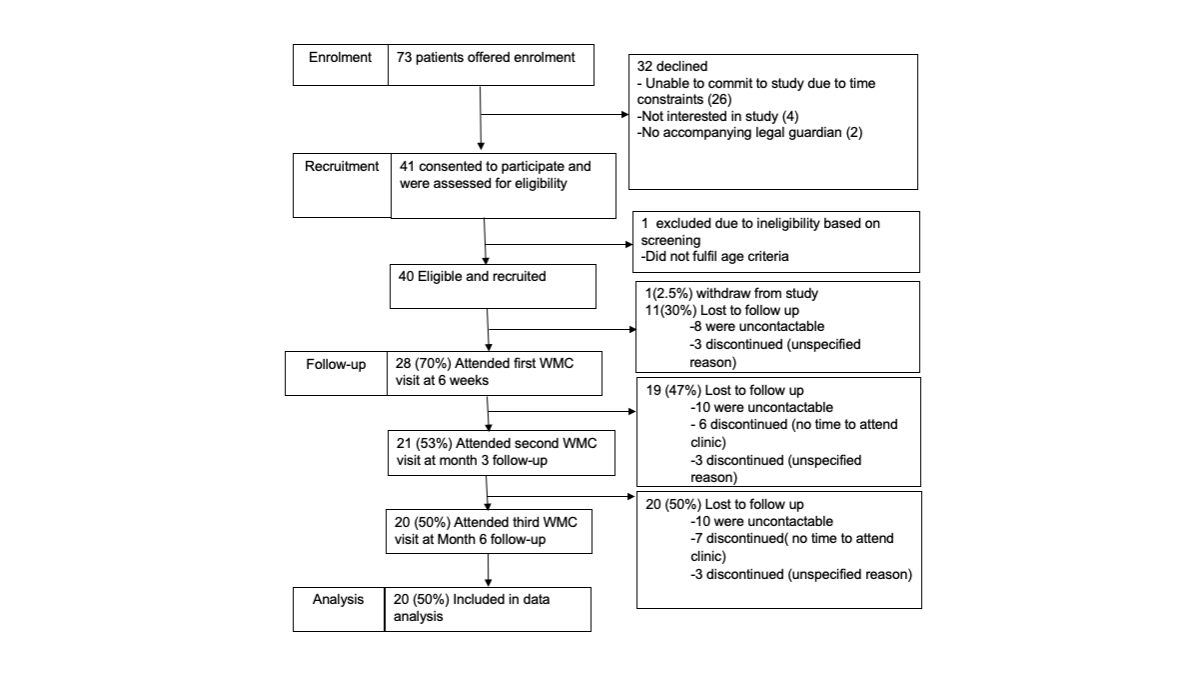
CONSORT (Consolidated Standards of Reporting Trials) diagram showing the flow of participants through each stage of the trial. WMC: weight management clinic.

Of the 73 eligible participants, 40 (55%) were consented and enrolled in the study. The mean age of the participants was 13.8 (SD 1.7) years. Among this, 58% (23/40) of the enrolled adolescents were male, 45% (18/40) were Chinese, 33% (13/40) were Malay, and 13% (5/40) were Indian. Of 40 participants, 32 (80%) were referred to WMC as a result of opportunistic screening during medical visits for nonobesity-related conditions and 8 (20%) were referred specifically for obesity-related comorbidities. Moreover, 65% (26/40) of participants had a family history of metabolic diseases, and 83% (33/40) of accompanying parents were overweight or obese. Overall, 83% (33/40) of participants had household income less than the national median monthly household income of SGD 9500 (US $7125) [26], and approximately one-third had a monthly household income below SGD 2000 (US $1500). Half of the accompanying parents had a secondary school–level education or less. Other baseline characteristics of the participants are summarized in Table 1. There were no significant differences in baseline characteristics between those who completed the study and those who dropped out (Table 1). In total, 53% (21/40) of adolescents completed the 3-month assessment, and 50% (20/40) completed the 6-month assessment (Figure 2).

**Table 1:** Baseline characteristics of participants in the study (N=40).

Characteristics				Total (N=40)		Completers (n=20)		Drop-outs (n=20)		*P* value	
**Baseline characteristics**											
	Gender (male), n (%)			23 (58)		12 (60)		11 (55)		.79	
	**Ethnicity, n (%)**										.25
		Chinese	18 (45)		12 (60)		6 (30)				
		Malay	13 (33)		3 (15)		11 (55)				
		Indian	5 (13)		2 (10)		3 (15)				
		Other	4 (10)		4 (35)		0 (0)				
	**Weight category, n (%)**										.32
		Overweight	8 (20)		5 (25)		3 (15)				
		Obesity	32 (80)		15 (75)		17 (85)				
	Family history of metabolic diseases, n (%)			26 (65)		12 (60)		14 (74)		.37	
	Age (years), mean (SD)			13.8 (1.7)		14.6 (1.5)		13.4 (1.8)		.62	
	Body mass (kg), mean (SD)			81.2 (17.2)		83.8 (19.1)		78.6 (15.1)		.34	
	Height (cm), mean (SD)			161.9 (11.4)		163.3 (12.2)		160.4 (10.7)		.43	
	BMI (kg/m^2^), mean (SD)			30.7 (3.9)		30.6 (4.3)		30.3 (3.5)		.79	
	BMI z-scores, mean (SD)			2.07 (0.30)		2.05 (0.34)		2.09 (0.25)		.68	
	Waist-to-height ratio, mean (SD)			0.61 (0.06)		0.60 (0.06)		0.61 (0.05)		.80	
	Body fat percentage (%), mean (SD)			43.3 (5.9)		42.8 (5.8)		44 (6)		.53	
	**Blood pressure (mm Hg), mean (SD)**										
		Systolic	121 (13)		120 (13)		117 (10)		.26		
		Diastolic	69 (9)		68 (10)		67 (5)		.60		
**Accompanying parental baseline characteristics**											
	Age (years), mean (SD)			43.6 (5.3)		44.7 (5.2)		42.5 (5.2)		.19	
	Gender (female), n (%)			33 (82)		17 (85)		16 (80)		.68	
	**Weight status, n (%)**										
		Overweight	11 (28)		7 (35)		4 (20)		.57		
		Obesity	22 (55)		10 (50)		12 (60)		.57		
	**Marital status, n (%)**										.79
		Married parent	33 (82)		17 (85)		16 (80)				
		Single parent	7 (18)		3 (15)		4 (20)				
	**Highest education level, n (%)**										.11
		Secondary school (equivalent to 10 years of education)	21 (53)		8 (40)		13 (65)				
		Diploma	11 (27)		8 (40)		3 (15)				
		Bachelor’s degree and above	8 (20)		4 (20)		4 (20)				
	**Monthly household income (SGD; US $), n (%)**										.27
		Below 1500 (1125)	13 (33)		4 (20)		9 (45)				
		1500-4499 (1125-3374.25)	12 (31)		9 (45)		3 (15)				
		4500-7500 (3375-5625)	11 (26)		6 (30)		5 (25)				
		Not reported	4 (10)		1 (5)		3 (15)				

#### Engagement and Evaluation

**Figure 3:**
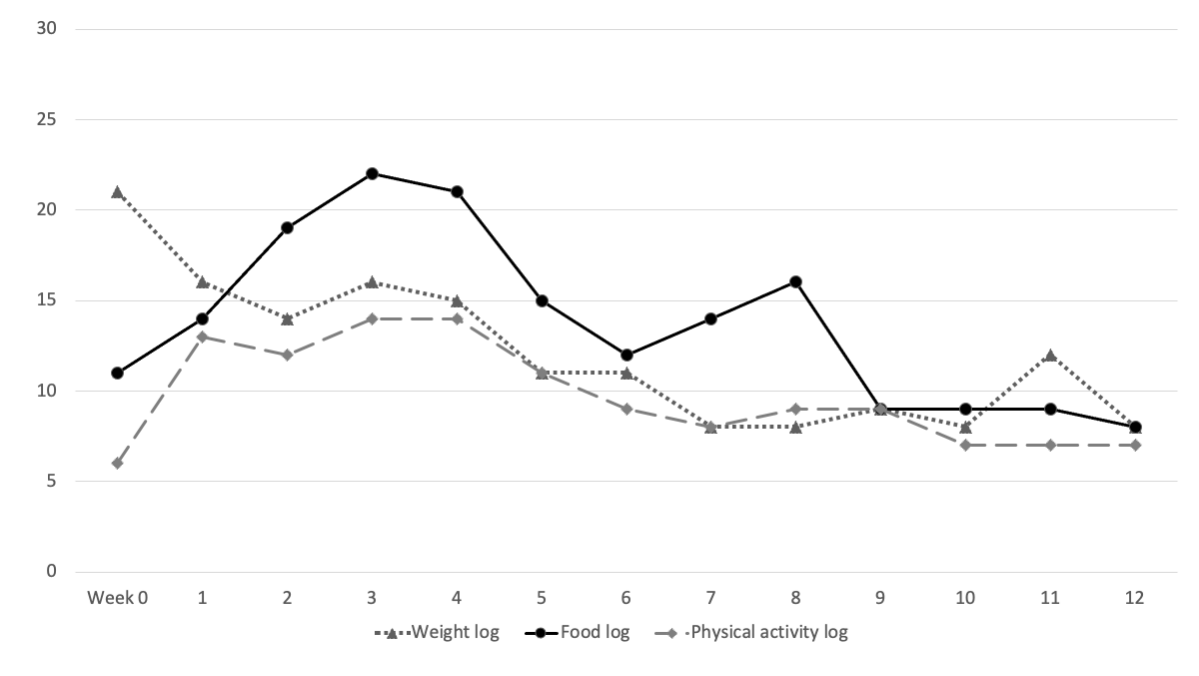
Number of participants who logged in at least one weight, food, or physical activity by week.

Overall, 83% (33/40) of participants completed at least 1 health coaching session. Participants completed a median of 7 (IQR 2-10) weekly sessions. Initial participant engagement across all Kurbo components was initially high but decreased over time (Figure 3). During the 12-week program, participants logged mean of 4.1 (SD 2.1) weight measurements. On average, participants tracked mean *green foods* 3.8 (SD 3.9) times and *red foods* 4.6 (SD 3.7) times and recorded physical activity 3.2 (SD 2.6) times each week. In addition, 18% (7/40) of participants did not engage in any health coaching session, weight, meals, or physical activity tracking.

**Figure 4:**
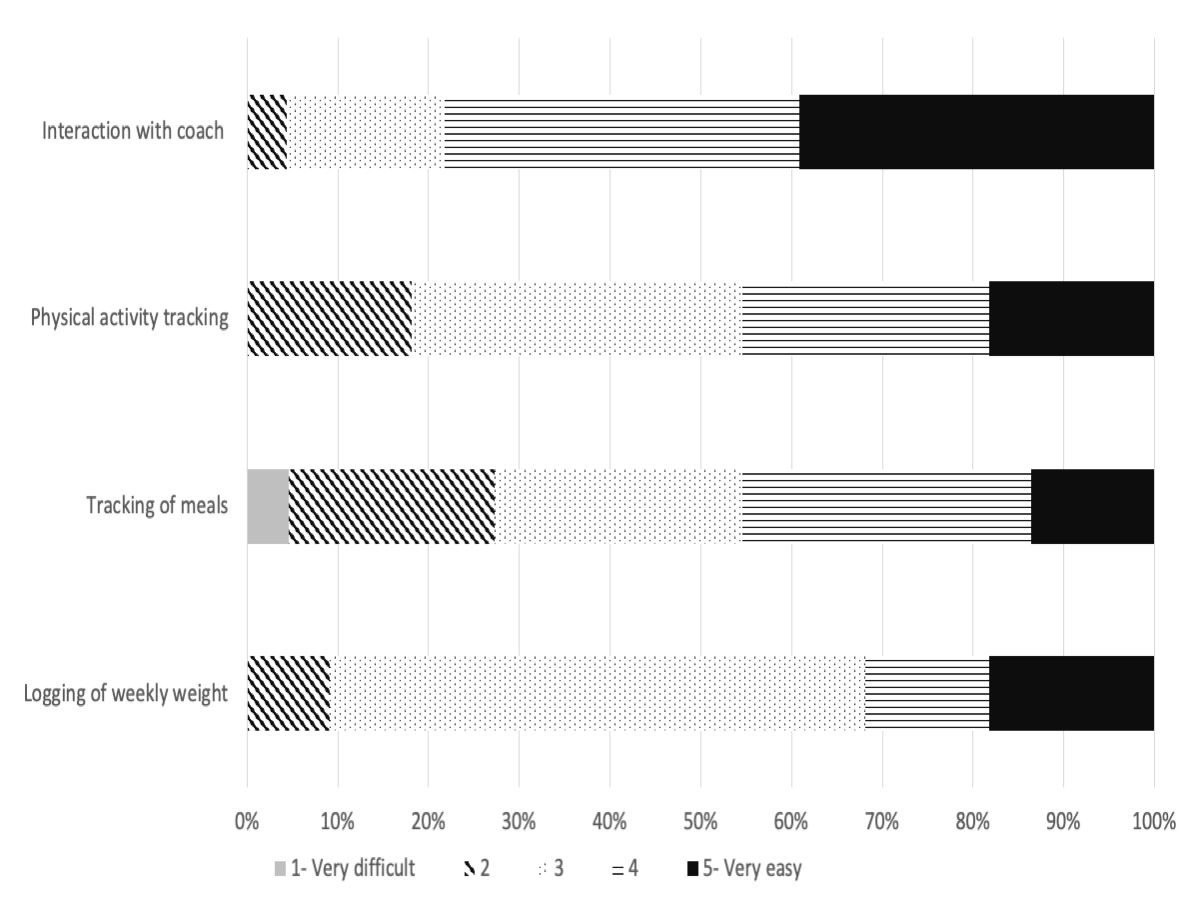
User-friendliness of the various components of Kurbo where 1=very difficult and 5=very easy.

**Figure 5:**
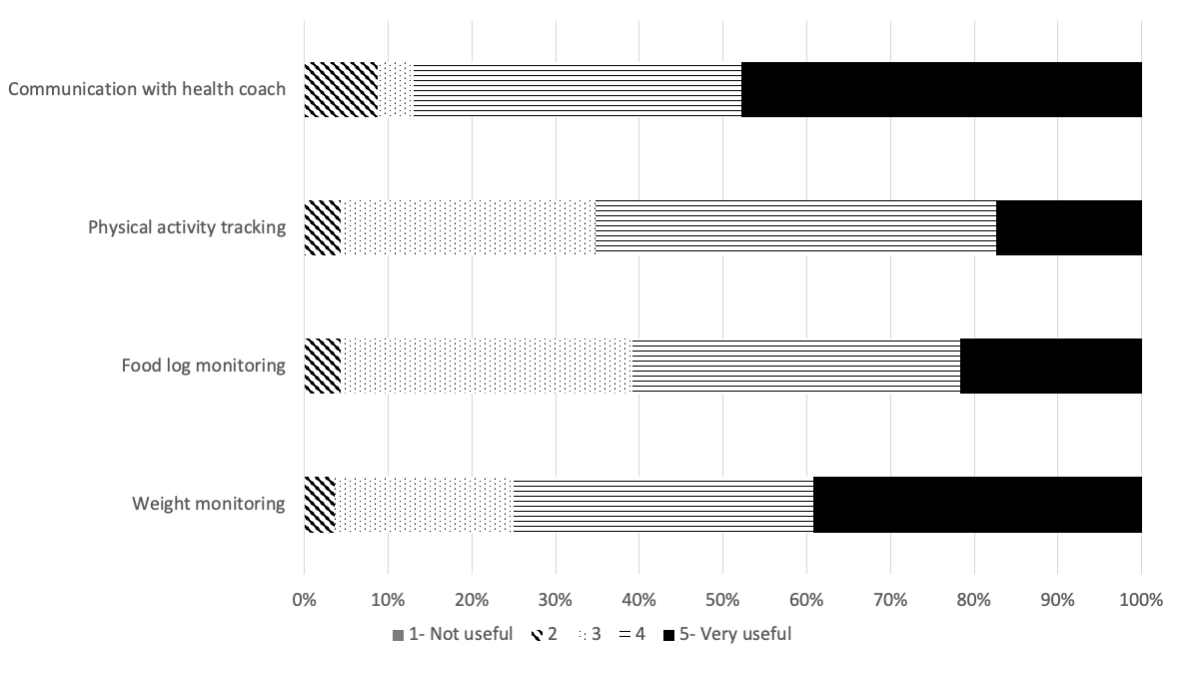
Usefulness of the various component of Kurbo where 1=not useful and 5=very useful.

A total of 23 participants completed the evaluation of Kurbo and individual components, as shown in Figures 4 and 5. Participants rated communication with the health coach as the most user-friendly and useful component. A total of 21 participants reported following the health coaches’ advice to a moderate-to-large extent. The participants did not report difficulties in using the app. Moreover, 18 participants rated the app as good to excellent, with 16 participants stating that they would recommend it to others.

### Effectiveness (3-Month Outcome) and Maintenance (6-Month Outcome)

#### Anthropometric and Blood Pressure Outcomes

There were no significant changes in BMI z-score (primary outcome) at either 3 or 6 months. However, there was a significant reduction in body fat percentage at both 3 months (−1.3%; 95% CI −2.5% to −0.2%; *P*=.03) and 6 months (−2%; 95% CI −3.6% to −0.4%; *P*=.02; Table 2). After adjusting for multidisciplinary weight management visits during engagement with Kurbo *P*, there was no significant difference in BMI z-scores at 3 months (*F*_27_=0.406; *P*=.53). No significant differences were detected at 6 months for blood pressure, waist circumference, or waist-to-height ratio (Table 2).

**Table 2:** Changes in adolescents’ anthropometric and blood pressure (N=40).

Variable	Baseline to 3 months (n=21)		Baseline to 6 months (n=20)	
	Value, mean (SD; 95% CI)	*P* value	Value, mean (SD; 95% CI)	*P* value
Body mass (kg)	2.7 (4.74; 0.5 to 4.8)	.02	3.59 (4.55; 1.45 to 5.71)	.002
BMI z-score	0.045 (0.15; −0.024 to 0.114)	.19	0.035 (0.14; −0.028 to 0.098)	.27
Waist circumference (cm)	1.1 (6.74; −1.9 to 4.2)	.45	0.3 (6.21; −2.6 to 3.2)	.84
Waist-to-height ratio	−0.003 (0.040; −0.20 to 0.015)	.75	0.004 (0.038; −0.014 to 0.022)	.67
Body fat (%)	−1.31 (2.54; −2.47 to −0.15)	.03	−2.0 (3.46; −3.6 to −0.38)	.02
Systolic BP^a^ (mm Hg)	−5.5 (9.21; −9.8 to −1.2)	.02	−2.1 (9.62; −6.8 to 2.5)	.35
Diastolic BP (mm Hg)	−4.2 (8.50; −8.1 to −0.2)	.04	−3.2 (8.47; −7.3 to 0.85)	.11

^a^BP: blood pressure.

#### Eating and Physical Activity Behaviors

The 3-day food diary revealed significant reductions in caloric intake at 3 months (mean −300, SD 456; 95% CI −576 to −24; *P*=.04) and 6 months (mean −332, SD 517; 95% CI 598 to −66; *P*=.02; Table 3) [5]. Moreover, the time spent in MVPA (minutes) at 6 months increased (mean 5.3, SD 4.8; 95% CI 0.88-9.75; *P*=.03; Table 3).

**Table 3:** Changes in adolescents’ health behavior and psychosocial parameters (N=40).

Variable			Baseline to 3 months (n=21)				Baseline to 6 months (n=20)		
			Value, mean (SD; 95% CI)		*P* value		Value, mean (SD; 95% CI)		*P* value
Total (kcal/day)			−300 (457; −576 to −24)		.04		−332 (518; −598 to −66)		.02
Servings of vegetables per day			−0.17 (0.50; −0.5 to 0.1)		.24		−0.0 (0.79; −0.4 to 0.4)		.99
Average moderate-to-vigorous physical activity per day (minutes)			1.47 (10.03; −4.3 to 7.2)		.59		5.3 (4.78; 0.88 to 9.75)		.03
**Adolescents** **Pediatric Quality of Life Inventory**									
	Total	2.4 (13.11; −3.1 to 7.9)		.38		1.4 (11.67; −4.2 to 7.0)		.61	
	Physical	4.0 (13.56; −1.7 to 9.8)		.16		1.0 (11.60; −4.6 to 6.6)		.72	
	Emotional	6.5 (23.34; −3.4 to 16.3)		.19		9.7 (20.58; −0.2 to 19.7)		.05	
	School	8.3 (18.28; 0.6 to 16.1)		.04		6.9 (13.52; 0.2 to 13.7)		.04	
	Psychosocial	6.3 (14.52; 0.2 to 12.5)		.04		6.5 (12.98; 0.2 to 12.7)		.04	
**Eating Pattern Inventory for Children**									
	Dietary restraint	0.033 (0.42; −0.15 to 0.21)		.72		0.00 (0.48; 0.23 to −0.23)		.99	
	External eating	−0.087 (0.82; −0.44 to 0.27)		.62		−0.19 (0.65; −0.50 to 0.12)		.22	
	Parental pressure to eat	0.116 (0.54; −0.12 to 0.35)		.31		0.018 (0.66; −0.30 to 0.33)		.91	
	Emotional eating	0.00 (0.79; −0.34 to 0.34)		.99		0.171 (0.67; −0.15 to 0.49)		.28	

#### Psychosocial Outcomes

At 3 months, adolescents’ self-reported quality of life improved in the school (mean 8.3; 95% CI 0.6-16.1; *P*=.04) and psychosocial (mean 6.3; 95% CI 0.19-12.5; *P*=.04) domains (Table 2). These improvements persisted for 6 months. There were no significant changes in eating patterns, including dietary restraint, external eating, parental pressure to eat, and emotional eating subscales.

## Discussion

### Principal Findings

This pilot study is one of the few studies to evaluate the implementation of a multicomponent mobile app as an early intervention before enrollment in an adolescent WMC. The Kurbo pilot was successful in reaching a low-income and racially diverse population. Although there was no significant reduction in BMI z-scores, there were significant improvements in fat percentage, total caloric intake, and quality of life, suggesting potential benefits of enrolment and the need for a more formal randomized trial.

Obtaining a reach of 58% is comparable with that of other pediatric obesity studies [27]. Moreover, the high percentage of minority, lower education, and low-income enrollees suggests that Kurbo is more likely to reach disadvantaged populations than traditional in-person programs. Given the high rates of obesity among children of lower socioeconomic status, this is a significant advantage of Kurbo.

Kurbo participants completed a median of 7 (IQR 2-10) coaching sessions. This level of engagement is considered very low (<10 hours of intervention time) based on the US Preventive Special Task Force criteria [28]. This may account for the lack of BMI changes among our participants. However, this lack of engagement is not unique to Kurbo, as other programs, including clinic-based programs, have shown similar levels of engagement [29-31].

Our results show that the maximal period of engagement with Kurbo occurred in the first 7 weeks, which corresponded to the period between the initial WMC referral and the first WMC clinic visit. This suggests that Kurbo may be helpful in engaging participants as an early intervention before the first WMC visit. Early engagement may account for the increase in WMC attendance at 6 months (20/40, 50% attendance) compared with our historical rate of 42.1% (51/121). The use of a mobile app as an early intervention also provided mutual benefits to both the health care provider team and Kurbo health coaches. The administrator platform allowed the multidisciplinary team to gain a better understanding of patient progress in health behaviors and weight before presenting to the clinic. This allowed for more targeted discussions about barriers that adolescents faced in the management of obesity and more efficient care. Kurbo health coaches were able to highlight any concerns that they faced during the health coaching sessions of the health care team.

A challenge with Kurbo as an early intervention was the high attrition rate. Although the study’s dropout rate of 50% (20/40) is less than the in-clinic rate of 57.8% (70/121), it suggests that strategies need to be crafted to reduce attrition if Kurbo is to be successfully used as an early-stage intervention. Further research on the reasons for attrition is recommended.

Despite the lack of changes in BMI z-scores, a significant improvement in quality of life is an important finding. Quality of life among adolescents with overweight is lower than that among normal-weight peers [25] and youth with other chronic conditions [32]. Improvement in quality of life has been reported with participation in other obesity treatment programs, even in children who do not achieve significant weight loss [33]. Given the prevalence of weight-based victimization experienced by adolescents with obesity in school [34], the improvement in the school dimension of quality of life is promising. Improvement in quality of life is critical, as it has been associated with improved long-term health indicators, with lower use of health care resources and greater long-term weight reduction [35]. To the best of our knowledge, improvement in self-reported quality of life has not previously been reported with the use of a mobile app to address adolescent obesity, which should be validated in future research.

Reassuringly, our study found no measured increase in disordered eating behaviors, as measured by the EPI-C. On the basis of these results, the integrated model of a mobile app with multidisciplinary adolescent obesity management is unlikely to increase disordered eating behaviors despite concerns for the development of disordered eating habits with the use of mobile apps [36].

### Limitations

This study had several limitations. The study had a small sample size and a high attrition rate. Second, as this was a feasibility study, the study did not include a control group.

### Conclusions

In this pilot study, the use of the Kurbo mobile app as an early intervention before a multidisciplinary clinical care for adolescent obesity treatment is feasible in a low-income and ethnically diverse Asian population. Although there was no significant change in the BMI z-score, Kurbo showed promise in improving quality of life and reducing body fat percentage and total caloric intake. Given the promising outcomes in several dimensions, further research using more rigorous trial designs should be conducted to evaluate the effects of Kurbo as part of an early, stepped care intervention for adolescents with obesity.

## Abbreviations

EPI-C: Eating Pattern Inventory for Children
KKH: KK Women’s and Children’s Hospital
mHealth: mobile health
MVPA: moderate-to-vigorous physical activity
WMC: weight management clinic
